# Mechanistic lessons learned from studies of planktonic bacteria with metallic nanomaterials: implications for interactions between nanomaterials and biofilm bacteria

**DOI:** 10.3389/fmicb.2015.00677

**Published:** 2015-07-17

**Authors:** Navid B. Saleh, Bryant Chambers, Nirupam Aich, Jaime Plazas-Tuttle, Hanh N. Phung-Ngoc, Mary Jo Kirisits

**Affiliations:** Department of Civil, Architectural and Environmental Engineering, The University of Texas at AustinAustin, TX, USA

**Keywords:** metal-oxide nanoparticles, reactive oxygen species, dissolved ion, physical disruption, EPS

## Abstract

Metal and metal-oxide nanoparticles (NPs) are used in numerous applications and have high likelihood of entering engineered and natural environmental systems. Careful assessment of the interaction of these NPs with bacteria, particularly biofilm bacteria, is necessary. This perspective discusses mechanisms of NP interaction with bacteria and identifies challenges in understanding NP–biofilm interaction, considering fundamental material attributes and inherent complexities of biofilm structure. The current literature is reviewed, both for planktonic bacteria and biofilms; future challenges and complexities are identified, both in light of the literature and a dataset on the toxicity of silver NPs toward planktonic and biofilm bacteria. This perspective aims to highlight the complexities in such studies and emphasizes the need for systematic evaluation of NP–biofilm interaction.

## Introduction

Nanomaterials show unique electrical, optoelectronic, physical, catalytic, and photoactive properties. Metallic nanoparticles (NPs), such as Ag (Chambers et al., 2014) and Cu (Shah et al., 2012; Arijit Kumar et al., 2014), and nano-scale metal-oxides, such as ZnO (Li et al., 2011a,b), TiO_2_ (Huang et al., 2008; Brunet et al., 2009; Simon-Deckers et al., 2009), and CuO (Heinlaan et al., 2008; Chang et al., 2012; Ivask et al., 2014), exhibit antimicrobial properties, making them useful for water treatment (Theron et al., 2008), odorless textiles (Simoncic and Tomsic, 2010), bandages (Lo et al., 2009; Chaloupka et al., 2010), and biomedical and dental implants (Chen and Schluesener, 2008; Allaker, 2010).

Substantial research has examined the impact of metal and metal-oxide NPs on planktonic (i.e., free-swimming) bacteria. These studies have identified key NP attributes related to NP–cell interaction and associated mechanisms of toxicity for planktonic cells, but the relevance of that literature to NP–biofilm interaction is unknown because the biofilm environment and the biofilm cells themselves are fundamentally different from their planktonic counterparts. This perspective discusses current knowledge on nano-bacterial interaction via underlying antimicrobial mechanisms, notes differences between NP interaction with planktonic and biofilm bacteria, and identifies challenges in NP–biofilm interaction as governed by the complexities of the bacterial biofilm.

## Differences between Planktonic and Biofilm Cells

**FIGURE 1:**
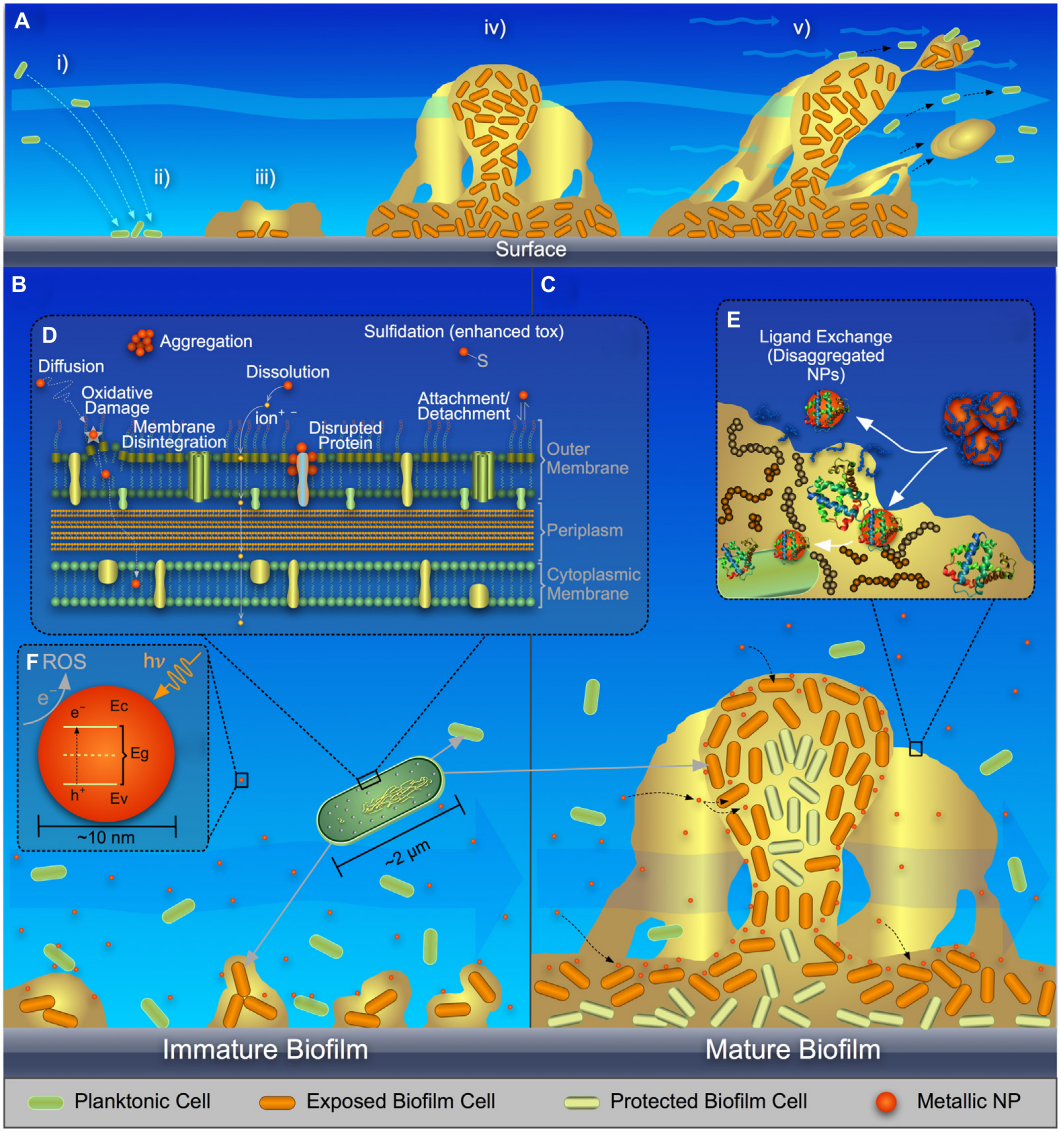
**Schematic representing (A) stages in biofilm formation and growth, (B) metal and metal-oxide nanoparticle (NP) interaction with planktonic bacteria and immature biofilm, (C) metal, and metal-oxide NP interaction and translocation in mature biofilm, (D) a close-up of NP interaction with a cell surface, (E) ligand-exchange process of NPs with extracellular polymeric substances (EPS) in a biofilm, and (F) band architecture of a metal-oxide NP showing reactive oxygen species (ROS) generation via electron transfer from the valence band (*E_v_*) to the conduction band (*E_c_*) and eventually to the surrounding fluid**.

Biofilms [i.e., collections of bacteria associated with a surface and surrounded by a matrix of extracellular polymeric substances (EPS)], are common in natural and engineered environments. Planktonic cells attach to a surface (**Figure 1Ai,ii**); an EPS matrix is produced (**Figure 1Aiii**) consisting of polysaccharides, proteins, DNA, and lipids (Flemming and Wingender, 2010); the biofilm matures (**Figure 1Aiv**) during which time the spatial distribution of EPS components can change (Ma et al., 2009); the biofilm acts as a viscoelastic fluid that can undergo detachment events (Stoodley et al., 2002; **Figure 1Av**).

Planktonic and biofilm cells exhibit key differences. (1) As compared to planktonic cells in a homogeneous environment, biofilm cells are exposed to different microenvironments depending on their location in the biofilm; for instance, biofilms can have gradients of electron donor and acceptor (Tang et al., 2011). (2) Many bacteria can sense surface contact (e.g., by inhibition of flagellar motion) and modulate their transcriptional patterns accordingly (reviewed in Wozniak and Parsek, 2014). For instance, the transcriptional patterns of *Campylobacter jejuni* shifted more toward iron uptake, oxidative stress combat, and membrane transport in biofilms as compared to planktonic cells (Sampathkumar et al., 2006). (3) Biofilms generally produce substantially more EPS as compared to planktonic cells, especially at low specific growth rates (Evans et al., 1994). Relatedly, distinct differences were found between the transcriptional profiles of *Pseudomonas aeruginosa* biofilm and planktonic cells, where the expression of EPS-related genes was an important hallmark of the biofilm (Dötsch et al., 2012).

Such differences between planktonic and biofilm cells will influence the impact of antimicrobial agents. As compared to planktonic cells, biofilm cells often show increased tolerance and resistance to traditional antimicrobial agents; such observations are linked with biofilm-specific pumps (Gillis et al., 2005), protection due to biofilm EPS (Billings et al., 2013), and the ability of biofilms to reduce the concentrations of reactive oxygen species (ROS; Nguyen et al., 2011; Khakimova et al., 2013). Thus, the antimicrobiality of NPs (i.e., non-traditional antimicrobial agents) is likely different between planktonic and biofilm cells.

## Key Mechanisms of Nano-Bacterial Interaction

Several major mechanisms have been proposed to explain the stress/toxicity to bacteria exposed to metallic NPs as reviewed by others (Nel et al., 2006; Manke et al., 2013; Reidy et al., 2013; von Moos and Slaveykova, 2014; Djurišić et al., 2015): (a) ROS-mediated oxidative stress, with lipid peroxidation and DNA damage; (b) dissolution of metal ions, which react with cellular components; (c) and physical disruption of the cell envelope. For planktonic bacteria (**Figure 1B**), these mechanisms are described as follows.

### Effects of ROS

ROS production, under the influence of photo- or chemical-activation, is a common stress/toxicity mechanism for bacteria exposed to metal and metal-oxide NPs [e.g., quantum dots (Lu et al., 2008), Ag (Choi and Hu, 2008), TiO_2_ (Li et al., 2012), CuO (Zhao et al., 2013; Laha et al., 2014), and ZnO (Li et al., 2012)]. ROS is an aggregate term that encompasses radical and non-radical forms of high energy chemical species, such as singlet oxygen, ^1^O_2_; superoxide anion, 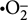; hydroxyl radical, •OH; and hydrogen peroxide, H_2_O_2_ (Thannickal and Fanburg, 2000; Finkel, 2001; Apel and Hirt, 2004). ROS can be formed as byproducts of aerobic metabolism (D’Autréaux and Toledano, 2007) and might act as regulatory molecules in prokaryotic cells (Finkel, 2001; Cabiscol et al., 2010). Cells have multiple pathways to limit ROS build-up (D’Autréaux and Toledano, 2007), but loss of cellular function can occur when this capacity is exhausted. As summarized in **Figures 1D,F**, metal and metal-oxide NPs can induce ROS outside the cell, at the cell membrane, and inside the cell (when NPs are internalized) by direct interaction with biomolecules in the environmental medium, the cell/outer membrane, and organic cytoplasmic components, respectively, or via similar interactions of dissolved metal ions with biomacromolecules (Park et al., 2009; Cabiscol et al., 2010; Dutta et al., 2012); recent studies of metal-oxide NPs have attempted to correlate conduction band-edge positioning with respect to cellular redox potential and the resulting ability to generate ROS (Zhang et al., 2012; Kaweeteerawat et al., 2015).

Extracellular or cell-surface ROS can compromise cellular integrity; membrane-leakage can be incurred via lipid peroxidation or protein modifications (Dutta et al., 2012). Intracellular ROS results in similar lipid peroxidation and protein modification, as well as DNA damage (Sies and Menck, 1992; Cabiscol et al., 2010; Laha et al., 2014).

### Effects of Dissolved Metals

Ion release from metallic NPs (**Figure 1D**), such as the release of Ag^+^, Zn^2+^, or Cu^2+^ from nano-scale Ag, ZnO, or CuO, respectively, is an important cause of the antimicrobiality of NPs (Marambio-Jones and Hoek, 2010; Ma et al., 2013; Chambers et al., 2014; Ivask et al., 2014). NP dissolution can occur outside the cell, at the cell surface, or within the cell. Dissolved metals can impact cellular functions, primarily via coordination and non-homeostasis (Chang et al., 2012). Chelation of metal ions with the chemical moieties of intracellular or extracellular ligands, e.g., oxygen, phosphorus, nitrogen, and sulfur functional groups, can alter biomolecule structure or function. For example, Ag^+^, known to dissolve from silver nanoparticles (AgNPs; Chambers et al., 2014), forms adducts with respiration enzymes, DNA, and membrane-associated proteins via thiol groups, thereby damaging cellular function (Feng et al., 2000; Matsumura et al., 2003; Lok et al., 2006; Wigginton et al., 2010). Relatedly, for the current study, we used live-dead staining with flow cytometry to show loss of membrane integrity in *Escherichia coli* with increased Ag^+^ exposure due to AgNP dissolution (Supplementary Figure S1).

### Physical Disruption of the Cell Membrane

As shown in **Figure 1D**, interaction of metal or metal-oxide NPs with the cell surface can result in chemically and physically induced toxicity (Pal et al., 2007; Okyay et al., 2015). The interaction of NPs with the outer membrane/lipopolysaccharide (LPS) for Gram-negative bacteria and the cell wall/membrane for all bacteria is dependent on local chemistry (e.g., NP coating, LPS composition, pH, and ionic strength). NPs are often coated to stabilize them in aqueous suspension, which typically introduces surface charge and inhibits nanoparticle dissolution/aggregation (Ghosh Chaudhuri and Paria, 2012; Li et al., 2013); Gram-positive and Gram-negative bacteria often are negatively charged in solution (Stendahl et al., 1977; Caputy and Costerton, 1984). The interaction between charged NPs and bacteria can be described via Derjaguin, Landau, Verwey, and Overbeek (DLVO) theory, which includes attractive van der Waals and repulsive electrical double layer forces. The surface functionality of the NPs can induce transport of these particles to and through the cell membrane via favorable electrostatic interaction (Feris et al., 2010; Suresh et al., 2012). Once the NPs undergo an interfacial journey to the inside of a cell, further chemical reactions can take place.

Nanoparticle shape is important in the disruption of the cell envelope. CuO nano-sheets and nano-spheres attached to bacterial cells, but nano-sheets produced more surface damage in Gram-positive bacteria and nano-spheres produced more surface damage in Gram-negative bacteria (Laha et al., 2014). In addition to acting as a localized source of ROS or dissolved metals outside a cell, NPs might cause direct physical stress to the cell membrane [e.g., due to sharp edges of ZnO nanorods (Okyay et al., 2015)]. Such physical disruption is impacted by the presence of dissolved organic matter, which coats NP surfaces and reduces the ability of NPs to injure cells (Zhao et al., 2013).

## Planktonic versus Biofilm Interaction with NPs

Many studies have evaluated the interaction between NPs and planktonic bacteria, but few have assessed NP–biofilm interaction. Planktonic cells present a very different interaction environment as compared to mature biofilms (**Figures 1B,C**). Planktonic cells in a homogenous environment have similar gene expression, metabolic activity, and EPS production (Mikkelsen et al., 2007). Previous studies focusing on traditional antibiotic challenges to bacteria found that the lower metabolic activity of biofilm cells can reduce the effectiveness of certain antibiotics (Walters et al., 2003). Some studies suggest that EPS plays an important role in limiting antibiotic diffusion through the biofilm (Stewart and Costerton, 2001), thus acting as a physical barrier. The well-studied effects of antibiotics on bacteria provide evidence of unique interactions with planktonic versus biofilm cells. NPs as antimicrobial agents present additional complexities due to their unique surface moieties, shape, size, and aggregation propensity. The following section presents current knowledge regarding the NP–biofilm interaction in light of the literature and our laboratory data.

### NP-Biofilm Interaction: State-of-Knowledge

Of the few studies that have evaluated the impact of NPs on biofilms, some have shown that biofilms, as compared to planktonic cells, have reduced susceptibility to NPs (Fabrega et al., 2009; Choi et al., 2010). Choi et al. (2010) found that biofilms were four times less susceptible to AgNP exposure than were planktonic cells. Similarly, as compared to their planktonic counterparts, biofilms of *E. coli, P. aeruginosa*, and *Serratia proteamaculans* have been reported to have up to 25 times greater tolerance to AgNPs stabilized with hydrolyzed casein peptides (Radzig et al., 2013). Starch-coated NPs reduced *P. aeruginosa* and *Staphylococcus aureus* biofilm growth but completely inactivated planktonic cells at the same AgNP concentrations (Mohanty et al., 2012). Nano-scale titania under UV irradiation has been shown to produce ROS and substantially decrease the growth and development of *Pseudomonas fluorescens* (Arroyo et al., 2014) and *Bacillus subtilis* (Dhandapani et al., 2012) biofilms. Dissolved ions from nano ZnO have been shown to suppress biofilm formation of wastewater biofilms (Hou et al., 2014) and of the opportunistic pathogens *Rothia dentocariosa* and *Rothia mucilaginosa* (Khan et al., 2014).

Both planktonic and biofilm cells can produce EPS, which has been shown to lower the diffusion rate of NPs (Peulen and Wilkinson, 2011). However, EPS production is much greater in biofilms as compared to planktonic cells (e.g., Hall-Stoodley et al., 2004; Ruas-Madiedo and de los Reyes-Gavilán, 2005; Kives et al., 2006). Although capsular EPS can provide some protection to planktonic cells from NPs (Hessler et al., 2012), its protective capacity is limited in a planktonic environment due to its relatively small quantity as compared to EPS in biofilms.

### Results: Impact of AgNPs on Planktonic versus Bofilm Bacteria

For the current study, we assessed the tolerance of *E. coli* and *P. aeruginosa* biofilm and planktonic cells to mercaptosuccinic-acid-capped AgNPs. AgNP synthesis and tolerance assays were conducted as described previously (Chambers et al., 2014). Biofilm and planktonic cells were grown simultaneously in a spinning-disk reactor either in the absence of AgNPs or in the presence of a subinhibitory concentration (15.6 μg/L) of AgNPs. Approximately equal amounts of biofilm and planktonic cells were retrieved and placed into microtiter plates; after a 5-h exposure to 0-500 μg/L AgNPs, viable cells were enumerated via plate counts.

**FIGURE 2:**
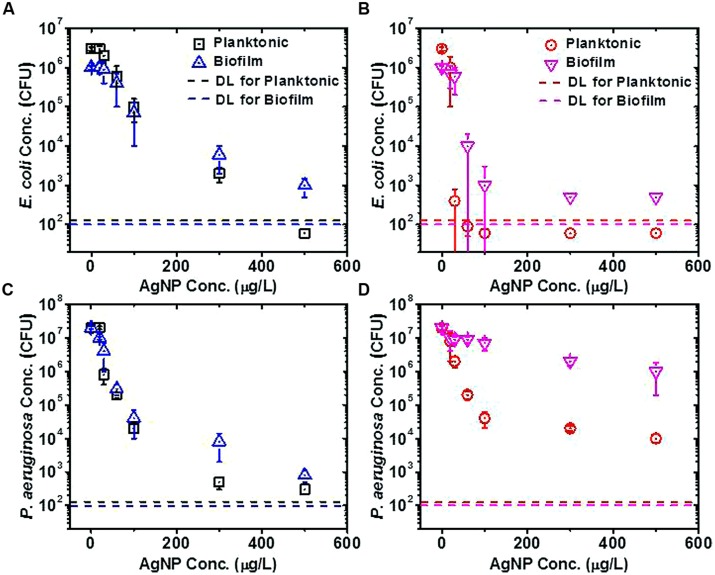
**Silver nanoparticle (AgNP) tolerance assays for planktonic and biofilm cells of *Escherichia coli* and *Pseudomonas aeruginosa*. (A)** *E. coli* cells without previous exposure to AgNPs, **(B)** *E. coli* cells with previous exposure to 15.6 μg/L AgNPs, **(C)** *P. aeruginosa* cells without previous exposure to AgNPs, **(D)** *P. aeruginosa* cells with previous exposure to 15.6 μg/L AgNPs. The detection limit (DL) for colony-forming units (CFU) in each sample was calculated by dividing the minimum CFU detectable on a plate (1 CFU) by the fraction of the total original sample volume plated for the planktonic or biofilm assay. Plate counts falling below the DL are plotted as one-half of the DL.

Biofilm and planktonic cells without previous exposure to AgNPs showed very similar tolerance to AgNPs upon exposure (*E. coli* in **Figure 2A** and *P. aeruginosa* in **Figure 2C**); thus, at each AgNP concentration, similar numbers of planktonic and biofilm bacteria survived the AgNP exposure. This was contrary to results from previous studies with traditional antimicrobial agents, where biofilms generally show higher tolerance to traditional antimicrobials than do planktonic cells (e.g., as for *P. aeruginosa* exposed to tobramycin; Kirisits et al., 2005).

However, the tolerances of biofilm and planktonic cells to AgNPs (for both *E. coli* and *P. aeruginosa*) diverged from one another when the cells were originally grown in the presence of a subinhibitory concentration of 15.6 μg/L AgNPs (see no substantial loss of viability at 15.6 μg/L AgNPs in **Figures 2A,C**). After having been grown previously in the presence of 15.6 μg/L AgNPs, biofilm cells generally showed greater tolerance to the subsequent AgNP exposure as compared to planktonic cells (**Figures 2B,D**), which is in keeping with what would be expected based on traditional antimicrobial studies. For planktonic and biofilm *E. coli* cells, the previous exposure to AgNPs did not provide an advantage to the cells when subsequently exposed to higher AgNP concentrations in the tolerance assay (compare **Figures 2A,B**); the previous exposure to the subinhibitory concentration weakened *E. coli*’s tolerance to subsequent AgNP exposures. On the other hand, for planktonic and biofilm *P. aeruginosa* cells, the previous exposure to AgNPs generally provided an advantage to the cells when subsequently exposed to higher AgNP concentrations in the tolerance assay (compare **Figures 2C,D**); thus, the previous exposure to the subinhibitory concentration improved *P. aeruginosa*’s tolerance to subsequent AgNP exposures. The differences between the *E. coli* and *P. aeruginosa* results suggest that these organisms employ different mechanisms to combat stress from AgNPs. These data also suggest that long-term exposure to low concentrations of NPs could render opportunistic human pathogens (e.g., *P. aeruginosa*) better able to withstand efforts to eradicate them with NP-based antimicrobial agents. While these conclusions pertain to AgNPs, generalizing these results to other metal NPs and bacteria requires investigation of bacterial stress response to NPs at a fundamental (e.g., transcriptomic) level.

## Future Challenges and Complexities in NP–Biofilm Interaction

The challenges in understanding NP–biofilm interaction arise from the inherent complexities of NPs and biofilms. NPs not only introduce variability in surface moieties, electronic structure, and chemical identity, but the interaction between NPs (i.e., homoaggregation) and the interaction of NPs with environmental surfaces (i.e., heteroaggregation) also complicate our understanding. The underlying antimicrobial mechanisms are not immune to these variations.

ROS production is correlated with conduction band position with respect to cellular redox potential (Zhang et al., 2012); however, nano-scale defects and metal doping will alter the band structure and shift the conduction band position, likely further complicating ROS-mediated toxicity. EPS on biofilms will likely coat NP surfaces, thereby altering electronic structure and ROS generation. Aggregation governs particle transport to planktonic cells and can influence the kinetics of particle mass delivery to bacteria. However, for biofilms, aggregation not only will influence transport and mass delivery but also will influence NP translocation through the biofilm. The absence of oxygen limits NP surface oxidation (Xiu et al., 2011); thus, the low oxygen microenvironments in deeper biofilm layers will likely limit the rate of dissolution of metal NPs, thereby reducing their antimicrobial activity.

Biofilm complexities will have a profound impact on NP–NP interaction or NP translocation through the biofilm, particularly due to EPS. Metallic NPs can deposit onto EPS polysaccharides (Ikuma et al., 2014). Thus EPS could act as a physical barrier to translocation of metal NPs through the biofilm. Studies have shown a reduction in NP diffusion in biofilms as compared to bulk solution (Habimana et al., 2011; Peulen and Wilkinson, 2011). EPS also might participate in ligand exchange, where the NP coating could be exchanged with EPS molecules, resulting in either alteration of NP aggregation or NP interfacial interaction with biofilm cells (**Figure 1E**).

Furthermore, effects of the classical mechanisms discussed for the interaction of NPs with planktonic cells might be exacerbated or ameliorated in biofilms. For example, the release of ROS and/or metal ions in biofilms likely is prolonged because of the longer residence time and localized interaction of the NPs with cells in the biofilm, as compared to their momentary interaction with planktonic bacteria (Peulen and Wilkinson, 2011). If metal ion release occurs, chelation of dissolved metals can occur with moieties in EPS (Kaplan et al., 1987; McLean et al., 1990); this would decrease the free metal ions available to attack cellular proteins and DNA. On the other hand, NP surfaces can undergo ligand exchange or passivation (**Figure 1E**) via chemical reaction with the molecules or moieties in biofilm EPS (Khan et al., 2011), which would decrease the release of metal ions. Further, entrapment of NPs within the biofilm might inhibit UV activation and associated ROS production, thereby reducing the antimicrobial activity of the NPs. Proteins and carbohydrate chains can react with ROS, thereby mitigating the effects of metal and metal-oxide NPs (Hessler et al., 2012).

Beyond the general advantage provided to biofilms by EPS, biofilm cells often have significant transcriptional differences as compared to planktonic cells (Sampathkumar et al., 2006; Dötsch et al., 2012). Numerous stress response elements are up-regulated in biofilms, which are closely associated with metal stress response, DNA repair, and metabolic stress. These systems might relieve stress caused by metal or metal-oxide NPs.

Systematic studies are necessary to understand NP interaction with and translocation through biofilms as well as stress modulation in biofilms exposed to NPs. Such studies need to carefully control NP attributes (e.g., aggregation, surface functionalities, electronic structure, size, and shape) and should assess NP interaction with biofilms at different stages of development. These controlled studies are needed to carefully decipher the mechanisms of interaction of NPs in the complex biofilm environment.

## Conflict of Interest Statement

The authors declare that the research was conducted in the absence of any commercial or financial relationships that could be construed as a potential conflict of interest.
