# Supplementary material for: Mechanistic lessons learned from studies of planktonic bacteria with metallic nanomaterials: implications for interactions between nanomaterials and biofilm bacteria
Source: Front Microbiol. 2015 Jul 17;6:677. doi: 10.3389/fmicb.2015.00677 (PMC4505144; doi:10.3389/fmicb.2015.00677)
Supplement: Supplementary file 1 [file Presentation_1.PDF]

Mechanistic Lessons Learned from Studies of Planktonic Bacteria with Metallic Nanomaterials: Implications for Interactions between Nanomaterials and Biofilm Bacteria

Navid B. Saleh, Bryant Chambers, Nirupam Aich, Jaime Plazas-Tuttle,
Hanh Nguyen Phung-Ngoc, and Mary Jo Kirisits

Supplementary Information

Materials and Methods

Bacterial cells were exposed to silver nanoparticles (AgNPs) using a previously described method; a detailed description of these methods is in Chambers *et al.* (2014)[1]. Briefly, *Escherichia coli* cells were cultured in a chemostat. Aliquots of cells were removed from the chemostat, vortexed for 30 seconds, and sonicated for 10 min in a bath sonicator (Branson 3510, Danbury, CT). *E. coli* cells were exposed to AgNPs in buffers with high ionic strength (Hμ=153 mM) and two different chloride concentrations (0 and 140 mM); these buffers are abbreviated as Hμ0 and Hμ140. The cells were incubated statically with 1 mg/L AgNPs in each buffer for 5 hours in the dark at 30˚C. Upon completion of exposure, cells were stained to prepare samples for the membrane permeability analysis.

Flow cytometry was used to examine membrane permeability of the cells and was conducted on a BD LSRFortessa (San Jose, CA). Samples (100 µL) were incubated with a mixture of Syto 9 (excitation 485 nm / emission 530 nm) and propidium iodide (excitation 485 nm / emission 630 nm) using a LiveDead® Baclight kit (Life Technologies; Grand Island, NY). Cells were incubated with both stains for 15 min in the dark at room temperature. Immediately after staining, cells were analyzed by flow cytometry using an autosampler with a 17-µL injection volume. Cells were binned first by side scatter and then examined by Syto 9 emission (FITC filter) and propidium iodide emission (PI filter). Results were analyzed using FlowJo (Version 9.8; Ashland, Oregon).

The dosed AgNPs (1 mg/L) dissolved to different degrees in the two tested buffers. Total aqueous silver was measured at the end of the 5-h exposure of AgNPs to *E. coli*. Aqueous silver was measured using centrifugal filters and quantified by Inductively Coupled Plasma Optical Emission Spectroscopy (ICP-OES) (Varian 710-ES, Mulgrave, Victoria, Australia). A detailed summary of the techniques used to prepare and analyze these samples is in Chambers *et al.* (2014)[1]. Theoretical equilibrium distributions of the aqueous silver were modeled using Visual MINTEQ (Version 3.0, John Gustaffason, KTH, Sweden).

Results

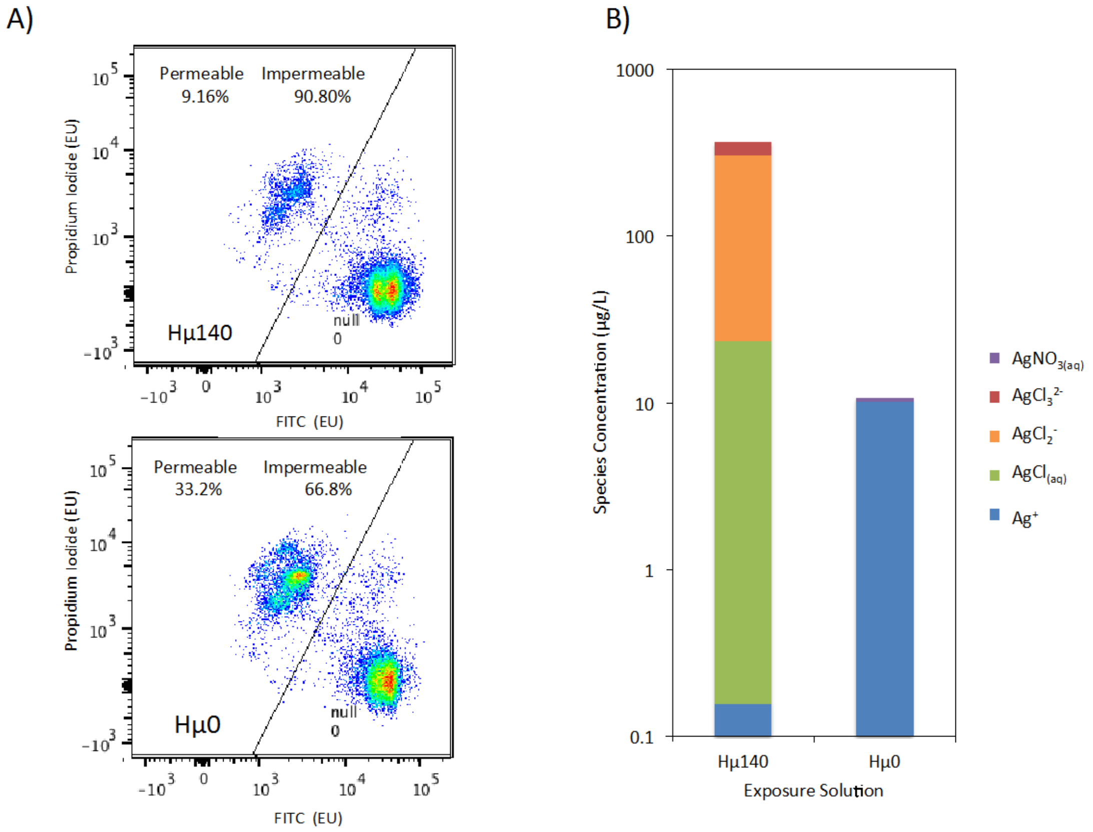

Figure S1. a) Permeability plots of *E. coli* cells measured using flow cytometry and Live/Dead® staining. Cells were examined using Syto 9 and a FITC filter (x-axis) and propidium iodide and its associated filter (y-axis) in emission units (EU). Cells with intact membranes (impermeable) stained only with Syto 9. Cells with compromised membranes (permeable) stained with both Syto 9 and propidium iodide, shifting the binned cells up and to the left in the plot. b) The theoretical equilibrium $AgCl_x^{(x-1)-}$ speciation of dissolved silver in a solution where 1 mg/L AgNPs were dosed to each buffer solution (Hμ140 and Hμ0). The resulting total dissolved silver was measured at 5 hrs via ICP-OES, and theoretical equilibrium speciation calculations were based on the total dissolved silver.

Increased membrane permeability (Fig. S1a) coincided with increased *E. coli* exposure to Ag^+ (Fig. S1b). While the buffer containing a higher chloride concentration, Hμ140, yielded greater overall dissolution of AgNPs (Fig. S1b), the silver-chloride complexes formed apparently had less impact on cell permeability as compared to Ag^+, which dominated in the Hμ0 buffer (Fig. S1b).

Supplemental References

1. Chambers, B.A., Afrooz, A.R.M.N., Bae, S., Aich, N., Katz, L., Saleh, N.B., and Kirisits, M.J. (2014). Effects of chloride and ionic strength on physical morphology, dissolution, and bacterial toxicity of silver nanoparticles. *Environ. Sci. Technol.* 48**,** 761-769.

.
